# Radiographic Identification of Foreign Bodies in the Maxillofacial Region: A Case Report and Review for Aesthetic Surgery Planning

**DOI:** 10.1155/crra/8870515

**Published:** 2025-09-03

**Authors:** Sekina Alimova, Yehya Tlaiss, Suzanne Youness, Ahmad Tleis, Basin Evgeny, Victor Truten, Tatiana Guseva

**Affiliations:** ^1^Department of Radiology, Federal State Budgetary Educational Institution of Higher Education, Russian University of Medicine, Ministry of Health of the Russian Federation, Moscow, Russia; ^2^Department of Ophthalmology, University of Balamand, Beirut, Lebanon; ^3^Department of Plastic Surgery, University of Balamand, Beirut, Lebanon; ^4^Department of Dental Surgery, Lebanese University, Beirut, Lebanon; ^5^Oncology and Plastic Surgery Department, Academy of Postgraduate Education Federal Medical-Biological Agency, Moscow, Russia; ^6^Department of Sports Medicine and Medical Rehabilitation, I.M. Sechenov First Moscow State Medical University (Sechenov University), Moscow, Russia

**Keywords:** aesthetic surgery, cone-beam computed tomography, foreign body detection, maxillofacial imaging, radiographic artifacts

## Abstract

Aesthetic facial and neck procedures increasingly rely on radiographic imaging for surgical planning and complication management. Cone-beam computed tomography (CBCT) offers high-resolution, three-dimensional imaging, but foreign materials such as cosmetic fillers and metallic implants can mimic pathology, generate artifacts, and complicate surgery. This case report with an integrated literature review highlights the radiographic identification of such materials and their implications for aesthetic procedures, illustrated by a patient case where CBCT revealed high-density inclusions in the submental region, correlating with intraoperative fibrosis and extended surgical time. CBCT proved superior in visualizing foreign bodies with lower radiation exposure than conventional CT, emphasizing the need for standardized imaging protocols and AI-driven artifact reduction to enhance diagnostic accuracy and optimize surgical outcomes.

## 1. Introduction

In recent years, there has been a significant increase in the number of cosmetic and surgical procedures aimed at addressing age-related changes, injuries, and defects or deformities of the face and neck [1]. Concurrently, the role of radiologic diagnostic methods has grown in importance for planning corrective procedures, evaluating treatment quality, and managing complications following aesthetic interventions. Clinicians now utilize the most appropriate imaging modalities tailored to their specific clinical needs.

Among available imaging techniques, cone-beam computed tomography (CBCT) is considered the most suitable imaging method for facial and neck aesthetic surgery, as it is currently the only modality that can be performed with the patient in an upright position [2]. Unlike MSCT, CBCT does not offer consistently standardized grayscale values, as its grayscale output is influenced by factors such as scatter, voxel size, and reconstruction algorithms. This limits its reliability in differentiating soft tissues or classifying foreign materials based on density. This capability is crucial because changes in body position, from standing to lying down, can significantly alter the distribution and appearance of soft tissues, which is particularly important in aesthetic evaluations [2]. Furthermore, CBCT offers three-dimensional visualization of the maxillofacial region, providing unparalleled accuracy in the planning of aesthetic surgeries.

However, CBCT also presents challenges, particularly when interpreting incidental findings. Foreign materials such as cosmetic fillers and implants can mimic pathological conditions like calcifications or tumors, complicating diagnostic accuracy and potentially leading to suboptimal surgical planning [3]. The absence of detailed medical histories, especially regarding prior cosmetic procedures or implanted materials in the face and neck, further complicates diagnosis and may extend operative times while increasing postoperative complication risks. Additionally, image artifacts caused by metallic materials, such as gold threads, add another layer of diagnostic complexity [3].

The integration of advanced imaging technologies, including CBCT and three-dimensional imaging, has markedly improved diagnostic accuracy and surgical planning in the maxillofacial region. Jaumotte et al. emphasize that these innovations enhance facial reconstruction outcomes by enabling the creation of anatomically precise and biocompatible implants [4]. Similarly, Bruehlmann et al. demonstrated that intraoperative CBCT use improves surgical outcomes by providing real-time verification of anatomical corrections, although challenges related to gender-based satisfaction variability were noted [5]. Despite these advances, the lack of standardized imaging protocols to differentiate cosmetic materials from pathological conditions remains a significant barrier to optimizing surgical planning and achieving superior clinical outcomes [3].

The aim of this case report with an integrated literature review is to address these challenges by describing the cosmetic materials identified during CBCT scans, supported by a synthesis of current evidence on imaging modalities in aesthetic surgery. Increasing awareness of the capabilities and limitations of radiologic diagnostics can help minimize diagnostic errors and improve both aesthetic and functional outcomes in patients who have undergone facial and neck aesthetic surgery.

## 2. Case Presentation

**Figure 1:**
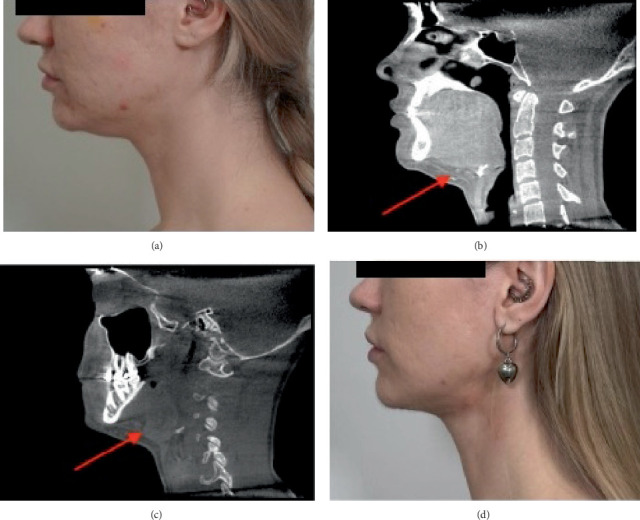
(a) Preoperative photograph showing submental fullness and blunted cervicomental angle. (b) Sagittal CBCT reconstruction demonstrating excessive accumulation of adipose tissue in the submental area, both subcutaneously and beneath the platysma muscle (red arrow). (c) CBCT image highlighting multiple high-density linear inclusions within the adipose tissue above the platysma (red arrow). (d) Postoperative photograph after surgical correction of the lower third of the face and neck.

A 34-year-old individual has a history of multiple cosmetic procedures, including repeated use of lipolytics and the drug Radiesse. The patient presented with complaints of soft tissue laxity in the neck and a blunted cervicomental angle. CBCT revealed an accumulation of adipose tissue in the submental area, both above and below the platysma muscle. Additionally, smoothness of the cervicomental angle was observed, attributed to platysmal sagging. Linear inclusions with bone-like density were identified predominantly above the platysma within the adipose tissue (Figure 1). These findings suggest the presence of scar tissue in these regions, potentially impacting the duration of the surgical procedure, the persistence of postoperative edema, and the overall surgical outcome. The patient was informed of these considerations during the preoperative consultation.

The patient underwent surgical correction of the lower third of the face and neck, which included liposuction of submental adipose tissue and lipectomy of subcutaneous fat beneath the platysma muscle via a submental approach. Additionally, excision of redundant skin from the anterior neck and submental region was performed. Intraoperatively, tissue dissection was complicated by significant fibrosis and the presence of foreign body inclusions, leading to increased bleeding and prolonged operative time. Postoperatively, the patient exhibited edema and irregularities in the subcutaneous tissue.

Imaging was conducted using a dental CBCT scanner, the “Volux55” (Genoray, South Korea), with a tube voltage of 60 kV and a field of view measuring 16 × 14 cm. The patient was positioned upright during imaging. Preoperative scans were subsequently imported into Miele-lxiv8.17.88 software for detailed analysis and data processing.

This study was conducted in accordance with the ethical standards of the institutional research committee. Ethical approval was obtained given the retrospective nature of the case report and anonymized data use. The patient provided informed consent for the use of their clinical and imaging data, with all identifying features removed to ensure privacy. Notably, patient images do not reveal identifiable facial features such as the eyes or other personal characteristics, maintaining confidentiality throughout the manuscript.

## 3. Discussion

CBCT has proven to be a valuable modality for detecting foreign bodies in the maxillofacial region, offering diagnostic accuracy comparable to MSCT but with a lower radiation dose and the advantage of upright patient positioning, which better reflects natural soft tissue distribution [6]. In this patient, CBCT enabled the identification of high-density linear inclusions within the submental adipose tissue, information that directly influenced surgical planning and patient counseling.

While ultrasound is effective for superficial foreign bodies, it would not have provided sufficient spatial resolution or depth evaluation in this case, where inclusions were located both above and below the platysma. MRI offers superior soft tissue contrast and can characterize filler materials, but its supine positioning alters gravitational soft tissue distribution, making CBCT the more suitable choice for preoperative evaluation in this aesthetic context [6, 7].

A comparative summary of imaging modalities is presented in Table 1.

## 4. Problems With Artifacts and Their Elimination

Metallic or dense cosmetic materials, such as gold threads, can produce beam-hardening artifacts on CBCT that obscure surrounding anatomy or mimic pathology [8]. In this case, mild streak artifacts were observed but did not prevent identification of foreign bodies or assessment of surgical planes. Recent advances in artificial intelligence (AI)–based image processing show potential for reducing metal artifacts and improving diagnostic clarity [9], which may further enhance preoperative planning in similar cases.

The differential imaging appearances of common cosmetic materials are summarized in Table 2.

Artifacts generated by metallic foreign bodies remain a significant challenge in CBCT imaging, potentially mimicking pathological lesions or obscuring relevant anatomy. Recent advances in AI-based image processing show promise in reducing metal artifacts, improving visualization, and enhancing diagnostic accuracy [9]. AI algorithms can differentiate true anatomical structures from noise generated by metallic implants, which facilitates better surgical planning and potentially reduces operative times.

Clinically, awareness of these foreign materials and their radiographic appearances can influence surgical strategy. For instance, recognizing extensive fibrosis or scar tissue related to filler materials preoperatively may prepare the surgeon for longer operative times, increased bleeding risk, and altered healing patterns. Preoperative CBCT imaging thus aids in informed surgical planning, patient counseling, and anticipation of possible complications, contributing to improved outcomes in aesthetic facial and neck surgeries.

## 5. Limitations

CBCT in this case allowed accurate three-dimensional localization of foreign materials but could not provide standardized density measurements, limiting differentiation between fibrosis and other dense inclusions. MRI could potentially offer better soft tissue characterization, but its limitations in accessibility, positioning, and cost made it less practical here. Ultrasound remains a useful adjunct for superficial lesions but would not have adequately visualized the deeper and bone-adjacent inclusions seen in this patient.

## 6. Recommendations

Standardized protocols should be developed to eliminate the variability of CBCT imaging and improve diagnostic consistency. They should include optimized visualization parameters for various foreign materials and special methods for reducing artifacts [8]. Valizadeh et al. emphasized that, although CBCT has advantages over CT in terms of radiation dose, clear recommendations are needed to maximize its use and ensure consistency in clinical practice [6]. Such protocols would increase the accuracy of diagnosis and contribute to more effective clinical decision-making.

## 7. Future Directions

The introduction of AI tools into CBCT workflows is an exciting stage. In addition to reducing artifacts, AI can help in the automated identification of foreign materials, which will further simplify the diagnostic process. In addition, comparative studies are needed to evaluate the cost-effectiveness of CBCT and its impact on patient treatment outcomes compared to other imaging methods [10]. Prospective studies involving large groups of patients are needed to confirm these approaches and develop evidence-based recommendations for the use of CBT in maxillofacial surgery.

## 8. Conclusion

Preoperative CBCT imaging plays an important role in detecting foreign materials in the head and neck region, providing surgeons with critical information that can influence surgical planning and timing. Awareness of the presence and location of cosmetic fillers, implants, and other foreign bodies helps anticipate potential complications such as fibrosis, scar tissue formation, and prolonged operative times. Although CBCT has limitations in soft tissue characterization, its advantages, including lower radiation dose and upright patient positioning, make it a valuable tool in aesthetic surgery planning, particularly when MRI is not feasible. In cases where radiation exposure must be minimized, ultrasound serves as a safe and effective first-line modality, particularly for superficial lesions or when dynamic, real-time imaging is beneficial. Clear communication with patients about these findings and potential surgical risks is essential to optimize outcomes. Further research and standardized imaging protocols are needed to enhance diagnostic accuracy and improve clinical decision-making in this evolving field.

## Figures and Tables

**Table 1:** Summary of the different imaging modalities.

**Imaging modality**	**Advantages**	**Limitations**	**Clinical use in aesthetic surgery planning**
Cone-beam CT (CBCT)	Low radiation, 3D imaging, upright position	Limited soft tissue contrast, no Hounsfield units	Detailed 3D bone and foreign body visualization, planning
Conventional CT	Good bone and soft tissue contrast, Hounsfield units	Higher radiation, supine position	Detailed tissue differentiation but higher dose
MRI	Superior soft tissue contrast, multisequence imaging	Expensive, long scan time, contraindications, supine position	Material characterization, soft tissue assessment
Ultrasound (US)	Real time, radiation-free, inexpensive, widely accessible	Limited depth and spatial resolution, no 3D imaging	Initial evaluation, superficial foreign body detection, and real-time guidance, especially in radiation-sensitive patients

**Table 2:** Differential diagnoses of common cosmetic materials on imaging.

**Material type**	**Imaging appearance (CBCT/CT)**	**Common location**	**Clinical implications**
Hyaluronic acid fillers	Low density, sometimes not visible	Subcutaneous tissue	Usually resorb over time
Calcium hydroxyapatite (Radiesse)	High density, granular calcifications	Soft tissue, muscle layers	May cause fibrosis or granulomas
Metallic implants (gold threads)	Very high density, beam-hardening artifacts	Subcutaneous or intramuscular	Can obscure imaging, complicate surgery
Barbed sutures	Linear high-density lines	Soft tissue	May require removal during surgery

## Data Availability

The data that support the findings of this study are available from the corresponding author upon reasonable request.
